# Quality of life of type 2 diabetes mellitus patients in Ramallah and al-Bireh Governorate–Palestine: a part of the Palestinian diabetes complications and control study (PDCCS)

**DOI:** 10.1007/s11136-020-02733-w

**Published:** 2021-03-02

**Authors:** Anna Katharina Tietjen, Rula Ghandour, Nahed Mikki, Lars Jerdén, Jan W. Eriksson, Margareta Norberg, Abdullatif Husseini

**Affiliations:** 1 University of Lübeck, Ratzeburger Allee 160, 23562 Lübeck, Germany; 2 Epidemiology Unit, Said Khoury Building for Development Studies, Institute of Community and Public Health, Birzeit University, P.O.Box 14, Birzeit, Palestine; 3 Epidemiology Unit, Said Khoury Building for Development Studies, Institute of Community and Public Health, Birzeit University, P.O.Box 14, Birzeit, Palestine; 4 St. John Eye Hospital, Sheikh Jarrah, P.O.Box 19960, 91198 East Jerusalem, Palestine; 5 School of Education, Health and Social Studies, Dalarna University, 791 88 Falun, Sweden; 6 Dept of Medical Sciences, Uppsala University, 751 85 Uppsala, Sweden; 7 Department of Epidemiology and Public Health, Umeå University, 901 87 Umeå, Sweden

**Keywords:** ADDQoL, Type 2 diabetes, Quality of life, Health status, Primary health care clinics, Palestinian diabetes complications and control study

## Abstract

**Purpose:**

Type 2 diabetes mellitus (T2DM) is a considerable impact on physical health as well as on emotional and social wellbeing. This study aimed to investigate the quality of life and its associated factors among Palestinians with T2DM.

**Methods:**

A cross-sectional study including 517 patients (68% female) was conducted in eleven primary health care clinics located in Ramallah and al-Bireh governorate of the West Bank. To assess socio-demographic data, risk factors and diabetes control, interviews, physical examinations, anthropometric measurements, and blood and urine tests were performed. The validated Arabic version of the Audit of Diabetes-Dependent Quality of Life (ADDQoL) questionnaire was carried out on all patients to measure Quality of Life (QoL). A multivariable regression analysis was performed.

**Results:**

The average weighted impact (AWI) score was −3.38 (95% CI: −3.55 to −3.21, range: −9.00 to 0.12). This indicates that diabetes was perceived as having a considerable negative impact on the quality of life. The life domains ‘freedom to eat’, ‘physical activities’, and ‘work-life’ were the most negatively impacted. Males and individuals living with diabetes for a prolonged time were associated with a more significant negative impact on quality of life.

**Conclusion:**

The study showed that diabetes generally had a negative impact on QoL and identified the demand for diabetes management programs tailored to patient needs and different patient groups, as well as health policies that put patients in the center of diabetes care.

**Supplementary Information:**

The online version of this article (10.1007/s11136-020-02733-w) contains supplementary material, which is available to authorized users.

## Introduction

Diabetes mellitus is of significant public health concern as it has dramatically risen in prevalence and has enduring complications on patients and their quality of life. In the Global Burden of Disease Study 2017, it was estimated that worldwide about 476 million people live with diabetes mellitus, of whom 463 million live with type 2 diabetes mellitus [1]. People in the Middle East and Palestine face an increasing prevalence of diabetes [2]. Based on estimates using 88 papers, including T2DM papers published between 1980 and 2015 from Arab states, the mean prevalence was estimated at 16.2% [3]. In Palestine, the prevalence is projected to increase from 18.4% in 2015 to 21.5% in 2030 [2]. Palestine is in epidemiological transition with diabetes emerging as one of the leading causes of morbidity and mortality in the region [4, 5]. Diabetes mellitus has increased from the 10th ranking cause of all deaths in 2005 to the 5th ranking cause of all deaths in 2018 [6, 7].

Diabetes is a metabolic disorder associated with chronic hyperglycemia that puts patients at risk of micro- and macrovascular complications, which may lead to high morbidity and mortality [8]. Clinical presentation, disease progression, and disease management vary from patient to patient considerably. However, it is important to underline that all patients experience a substantial impact on the different aspects of life. To live with T2DM means that one has to manage a lifelong disease every day by eating a healthy diet, being physically active, quitting smoking, visiting the doctor regularly for check-ups, monitoring blood glucose levels, and taking medicines if prescribed. This commitment to diabetes self-management may constrain patients’ life which could be a burden to one’s emotional and social wellbeing as well as to one’s economic status as prescribed medicines and regular doctor check-ups cost money and interrupt work routines. Beyond this, another burden to social and emotional wellbeing could be physical impairments due to T2DM such as pain, amputation and loss of vision. Currently, there is no universally accepted definition of quality of life to measure the social and emotional wellbeing as it is a multidimensional, subjective, and dynamic construct [9]. In simple terms, it is an individual evaluation of how good or bad one’s life is, which is subject to change and influenced by various factors [9].

A study on 1008 Palestinians involving 53% ‘healthy’ participants from the general population and 47% of patients drawn from health services indicated that the Quality of Life (QoL) is significantly worse in Palestine among healthy participants and patients compared to other countries as nearly 26% of the population in the occupied Palestinian territory reported ‘poor’ or ‘very poor’ QoL, compared to roughly 11% of the pooled population in the WHO International Field Trial, including all countries [10]. The authors pointed at the entrenched political conflict and chronic exposure to generational violence as the potential cause for the low QoL among the Palestinian population. Men, older adults, and those less educated showed to be affected the most compared to their counterparts. The authors proposed that a deeper understanding of QoL determinants is needed to fully elucidate the impact of the deep-rooted conflict on the wellbeing of people in Palestine [10]. Another study on Palestinian patients with T2DM (*n* = 385) using the Arab version of European Quality of life scale found that older age, being unemployed, and presence of comorbidities were associated to lower health-related QoL and suggested in order to improve QoL more attention should be placed on the elderly’s health and economic status [11]. The Audit of Diabetes-Dependent Quality of Life questionnaire (ADDQoL) is a disease-specific instrument that reacts more sensitively to subtle changes and may elucidate differences between subgroups [9, 12, 13]. The ADDQoL is the most frequently translated and widely used diabetes-specific QoL measure [13]. Thus, it allows a comparison to various countries. Two systematic reviews elucidated good psychometric properties of the ADDQoL [9, 13]. The questionnaire encourages patients to evaluate the impact of diabetes on different domains of their life, i.e. enjoyment of food, work-life, personal relationships, and physical appearance. Additionally, the patients assign every life domain importance and choose for selected domains ‘non-applicable’. Therefore, the ADDQoL is based on aspects of life that are of relevance to the patients. This paper is part of the Palestinian Diabetes Complications and Control Study (PDCCS) [14] and aims to investigate the quality of life among Palestinians with T2DM in Ramallah and al-Bireh as well as associated demographic and clinical risk factors using the ADDQoL.

## Methods

### Study design

This was a cross-sectional study on the quality of life of T2DM patients in Ramallah and El-Bireh governorate enrolled from eleven primary health care clinics offering diabetes care. The PDCCS was conducted between March and May 2012. The study design, patient enrolment, study sample size calculation, and data collection methods were published previously in details [14]. The aim of this study was to investigate the quality of life and its associated demographic and clinical risk factors among patients with T2DM. The ADDQoL questionnaire (Arabic version) was used to measure the individual’s perception of the impact of diabetes on their quality of life (Appendix 1). Patients of every age with T2DM using routine diabetes services at the selected clinics were eligible. Exclusion criteria included the following: T1DM, pregnancy, and inability to communicate.

### The ADDQoL-19

The validated Arab version of the ADDQoL-19 questionnaire was requested from the ADDQoL developers who approved its use in the current study [15]. The structural analysis, as well as the Cronbach’s α coefficient of internal consistency (*α* = 0.94), supported the validity and reliability of the ADDQoL-19 Arabic version and backs up the existing validation of the ADDQOL-19 Arab version (Appendix 2 and 3). The ADDQoL-19 consists of two overview items, which measure the general quality of life *“In general, my present quality of life is:”* from excellent (3) to extremely bad (−3) on a 7-point scale and the diabetes-dependent quality of life *“If I did not have diabetes, my quality of life would be:”* very much better (−3) to worse (1) on a 5-point scale. Additionally, 19 domain-specific items assess the impact of diabetes on specific aspects of life using a 5-point scale *“If I do not have diabetes, i.e. [my freedom to eat] as I wish to be:”* very much better/greater (−3) to worse/less (1) as well as the importance of the specific life domains *“[My freedom to eat] as I wish is:”* very important (3) to not at all important (0). Five life domains had a *“not applicable”* option. The impact was weighted by importance for each life domain by multiplication. The weighted impact ranges from −9 (maximum negative impact of diabetes) to + 3 (maximum positive impact). The weighted impacts per individual were averaged over the domain-specific items, which resulted in an average weighted impact (AWI). A lower AWI reflects a poorer quality of life, while a greater AWI reflects a better quality of life.

### Clinical variables

Body mass index (BMI) was stratified according to WHO standards in obese (BMI ≥ 30) and non-obese [16]. Hypertension was defined as having a blood pressure measurement ≥ 140 mmHg for SBP and/or ≥ 90 mmHg for DBP or being on hypertensive medication. It was stratified in having no hypertension, having controlled hypertension by medication (SBT < 140 and DBT < 90) and having uncontrolled hypertension with or without medication (SBT ≥ 140 and/or DBT ≥ 90). Dyslipidemia was defined as having abnormal levels of lipids in the blood and measured by high triglycerides levels (> 150 mg/dl) and low HDL cholesterol (men < 40 and women < 50 mg/dl) [17]. HbA1c related to glycated haemoglobin. HbA1c was stratified in controlled (< 7% or < 53 mmol/mol) and uncontrolled (≥ 7% or ≥ 53 mmol/mol). Diabetes duration was stratified in 1 to 10 years and above 10 years. Complications were stratified in having at least one complications including microvascular or macrovascular complications. The assessment is described elsewhere [14]. Self-reported variables were the following: physical activity which was defined as having performed at least once in the last seven days physical activity for 30 min continuously; smoking which was defined as currently smoking of any tobacco products on daily basis; the perceived capability to deal with diabetes on a 4-point scale; whether patients perceived having sufficient information on diabetes on a 4-point scale.

### Data analysis

Data were entered and analysed using IBM SPSS Statistics 22. For normally distributed continuous variables, mean and standard deviations (SD) were reported. For skewed continuous variables, median and lower and upper quartiles were reported. For categorical variables, frequencies and valid percentages were shown. The dependent variable, AWI, was normally distributed (Shapiro–Wilk test: *W* = 0.981, *p* < 0.001). To investigate correlations between AWI and the categories of the general QoL as well as the diabetes-specific QoL overview item, respectively, Bivariate Pearson Correlation was carried out. For regression modelling, variables were effect-selected conceptually based on literature. The investigated predictors were currently taking insulin [18–27], duration of diabetes [22, 24, 25], and having at least one complication [12, 18, 20, 21, 26, 28, 29]. All models were adjusted for age and sex. The predictors were investigated for correlation. Taking insulin and having complications were examined using Chi-squared test. For duration and the binary predictors, the t-test was used. After visual inspection of the scatterplots of the continuous predictors paired with the outcome, a linear regression model was chosen. Due to collinearity, the relative impact of the predictors on AWI was investigated individually in a model adjusted for sex and age. Subsequently, the predictors were entered in the full multivariable regression model adjusted for sex and age. The adjusted estimates and its 95% confidence intervals (CI) were reported.

### Ethics

Witnessed verbal informed consent was acquired from patients. The participants were allowed to withdraw from the study at any time without specifying any reasons. They were informed that their non-participation will not affect the services that they receive in the clinic. Interviews and tests were conducted in a private space to guarantee patients’ confidentiality. The blood and urine test results, as well as the eye exam results, were shared with the patients. The study was approved by the ethics committee at the Institute of Community and Public Health at Birzeit University as well as approved by health officials in the ministry of health, UNRWA, and participating NGOs.

## Results

### Description of the study sample

A total number of 517 patients participated in the study, of which 494 answered the ADDQOL (Table 1). The mean age was 58.1  ±  9.8 years. About two-thirds of them were female, and 77% of the participants were married. About half came from an urban setting, and almost 30% were educated with secondary school and higher. The clinical and biological measures of the patients obtained that 80% showed raised HbA1c levels, 62% were obese, 50% had uncontrolled hypertension, and 87% abnormal lipid levels. More than one-third of the patients had diabetes for longer than 10 years, and 76% had at least one complication. 40% of the patients took insulin, 85% self-reported not to smoke, and 30% were physically active. Almost 80% perceived that they were capable of dealing with T2DM in an excellent or good way and that the information given on diabetes management was excellent or good.

**Table 1:** Socio-demographic, clinical, and other characteristics of persons with T2DM in Ramallah and al-Bireh Governorate (*n* = 494)

Characteristic	Total N (%)
*Patient characteristics*	
Sex	
Female	351 (67.9)
Male	166 (32.1)
Age (years)	
Mean ± SD	58.1 ± 9.8
Below 60	290 (56.3)
60 and above	225 (43.7)
Education	
No formal education	89 (17.2)
Elementary school^a^	276 (53.5)
Secondary school and above^b^	151 (29.3)
Locality type	
Urban	254 (49.1)
Rural	161 (31.1)
Camp^c^	102 (19.7)
Marital status	
Currently married	398 (77.0)
Not Married^d^	119 (23.0)
*Clinical and biological measures*	
HbA1c, in %-points	
Median (IQR)	8.4 (7.2, 10.0)
Controlled (< 7)	98 (19.8)
Uncontrolled (≥ 7)	397(80.2)
BMI in kg/m^2^	
Median (IQR)	31.5 (28.1, 35.7)
Non-obese	193 (38.0)
Obese (BMI ≥ 30)	315 (62.0)
Hypertension	
Yes, uncontrolled	257 (50.2)
Yes, controlled by medication	146 (28.5)
No^e^	109 (21.3)
Dyslipidemia	
Yes^f^	429 (86.7)
No	66 (13.3)
*Medical conditions*	
Duration of diabetes in years	
Median (IQR)	8 (3, 14)
0–10	327 (63.4)
> 10	189 (36.6)
Any complication	
Yes, at least one^g^	365 (76.2)
None	114 (23.8)
*Diabetes management*	
Currently taking insulin	
Yes	209 (40.4)
No	308 (59.6)
Smoking status	
Smoker	78 (15.1)
Non-smoker or ex-smoker	439 (84.9)
Physical activity	
Yes	155 (30.0)
No	361 (70.0)
Perceived capability to deal with diabetes	
To a lesser degree and no, not at all	112 (21.7)
In a good way	274 (53.2)
In an excellent way	129 (25.0)
Perceived information given on diabetes management by health personnel	
Little and no	109 (21.2)
Good	257 (49.9)
Excellent	149 (28.9)

^a^Up to 10 years, ^b^11 years and above ^c^the camps included Alamari, Aljalazone and Deir Ammar ^d^not married included the following: never married 14%, divorced 10%, widowed 76%, ^e^normal BP was defined as ≤ 140 mmHg SBP or ≤ 90 mmHg DBP, ^f^ Dyslipidemia was defined as low HDL (men < 40, women < 50 mg/dl) and high triglycerides (> 150 mg/dl), ^g^excluding retinopathy

### ADDQoL

**Fig. 1:**
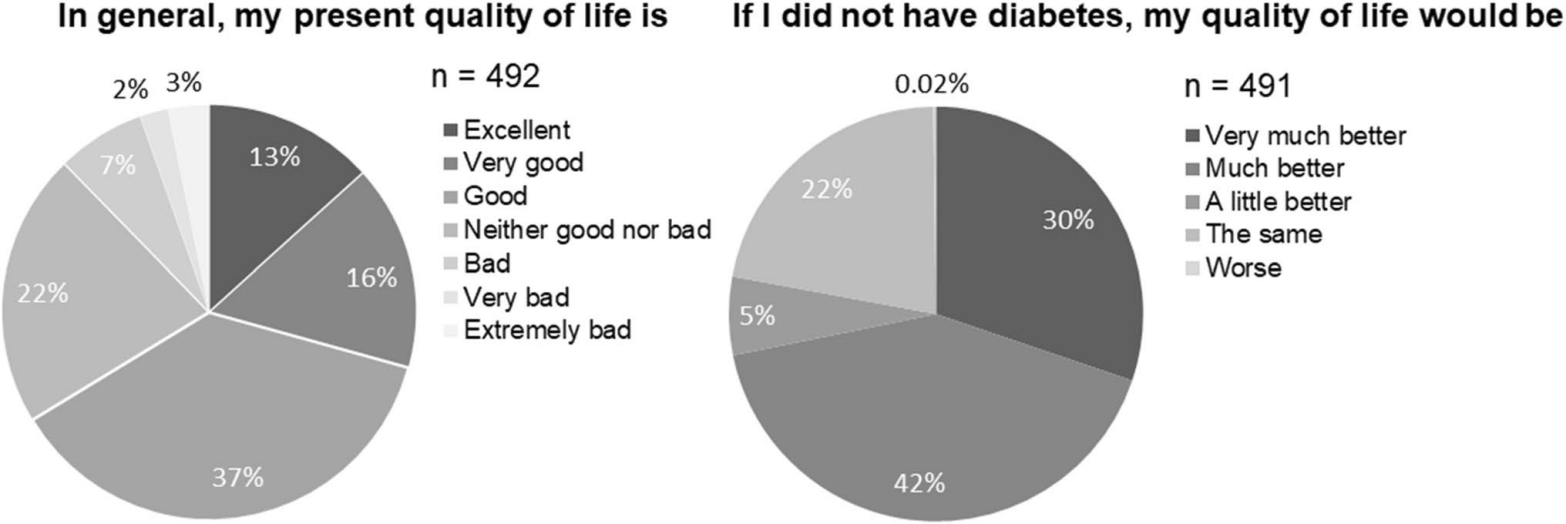
Pie charts of audit diabetes-dependent quality of life-19 (ADDQOL) results for the general and diabetes-specific overview items (Total population: *n* = 494)

In general, 66% of the Palestinians with T2DM perceived their quality of life as ‘excellent’, ‘very good’, or ‘good’ (Fig. 1). Regardless, 72% of the patients reported that their quality of life would be much or very much better if they did not have diabetes, while 22% perceived that the quality of life if they did not have diabetes would be about the same. The mean general QoL was between ‘good’ and ‘neither good or bad’ (mean ± SD = 0.879  ±  1.374) while the mean diabetes-dependent QoL was between ‘much better’ and ‘a little better’( mean ± SD = −1.797  ±  1.104).

**Fig. 2:**
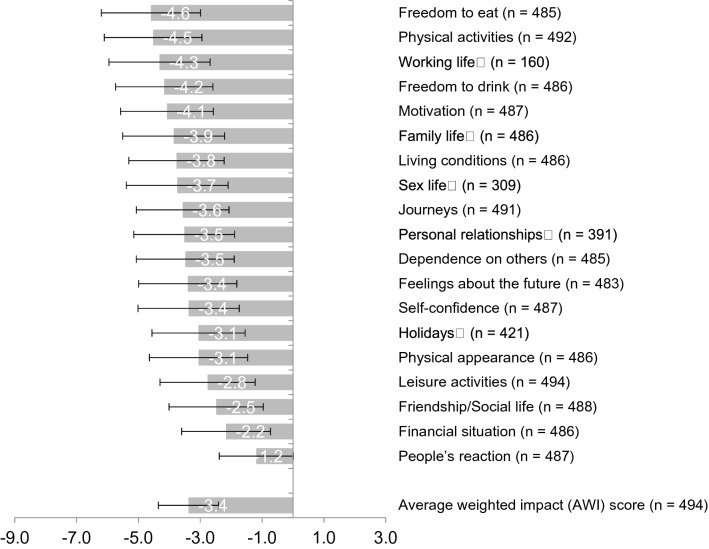
Results of the audit of diabetes-dependent quality of life-19 (ADDQOL) Arab version applied to 494 patients. The average weighted impact scores (AWI, mean±SD) are shown for the different life domains. The scale ranges from minimum −9 (impacted the most negative by diabetes) to a maximum 3 (impacted the least negative by diabetes). N differs as not all study participants filled in the ADDQOL questionnaire and some domains highlighted with a square had a ‘not applicable’ option

The mean average weighted impact score was −3.38 (95% CI for AWI: −3.55 to −3.21, range: −9.00 to 0.12), which indicates that diabetes is perceived as having a considerable negative impact on the quality of life (Fig. 2). Among the 19 domain-specific items, the patients with T2DM perceived ‘freedom to eat as I wish’ (AWI Mean ± SD: −4.60  ±  3.21), ‘physical activities as I wish’ (−4.53  ±  3.16), and ‘work-life as I wish’ (−4.32  ±  3.27) impacting their quality of life negatively the most. The patients perceived that diabetes had the least negative impact on people’s reactions (−1.19  ±  2.40) or their financial situation (−2.17  ±  2.87).

The patients assigned every life domain importance, which had an effect on the weighted impact ratings. For instance, it was observed that family life was of high relevance to the patients. It moved from rank 10 to 6 if impact was weighted by importance (Appendix 4). On the other hand, even though the domain holiday was perceived to be impacted by diabetes a lot, it was not of great importance to the patients as it shifted its rank from 9 to 14th if weighted by importance. The participants could choose for selected domains the ‘non-applicable’ (NA) option. The greatest use of NA was for working life (67.2% of 494 people), followed by sex life (34.9%), personal relationships (18.9%), holidays (14.4%), and family life (0.4%).

Bivariate Pearson correlation analyses showed that there was a correlation of AWI scores among the categories of the diabetes-dependent overview item (*r* = 0.445, *p* < 0.001). This indicated that people with diabetes perceived a more negative impact on their quality of life (having more negative AWI scores) and expressed more frequently that their life would be better without diabetes. The AWI was statistically not correlated to the general quality of life overview item (*r* = −0.085, *p* = 0.058).

### Regression analysis

The predictors were investigated for correlation. All predictors correlated highly: taking insulin and having complications ( χ^2^ = 18.2, *p* < 0.001), duration and taking insulin (*T* = −16.9, *p* < 0.001), and duration and complications (*T* = −4.722, *p* < 0.001). Looking at the sex- and age-adjusted estimates individually, currently taking insulin (95% CI: −0.992 to −0.302) and duration of diabetes (95% CI: −0.075 to −0.026) were predictors for a lower quality of life while complications was not (95% CI: −0.840 to 0.029) (Table 2).

**Table 2:** Results of the linear regression adjusted for sex and age individually for the predictors. The outcome was the average weighted impact (AWI) score obtained in the Audit of Diabetes-Dependent Quality of Life (ADDQOL) questionnaire (Arab version)

Model	Predictors	Adjusted for sex and age
Model 1	(Constant)	−3.514 (−4.778 to −2.250)
	Age (continuous)	−0.005 (−0.023 to 0.013)
	Sex Female Male	0.414 (0.049 to 0.778) ref
	Taking insulin currently Yes No	−0.647 (−0.992 to −0.302) ref
Model 2	(Constant)	−4.084 (−5.345 to −2.822)
	Age (continuous)	0.007 (−0.012 to 0.026)
	Sex Female Male	0.462 (0.100 to 0.824) ref
	Duration of diabetes (continuous)	−0.051 (−0.075 to −0.026)
Model 3	Age (continuous	−0.002 (−0.021 to 0.018)
	Sex Female Male	0.458 (0.070 to 0.845) ref
	Diabetes complications Yes, at least one No	−0.406 (−0.840 to 0.029) ref

Yet, entering the predictors in the full model adjusted for sex and age, taking insulin was not a predictor anymore (Table 3). Still duration of diabetes had an impact on AWI (95% CI: −0.070 to −0.008) as well as sex (CI 95%: 0.064 to 0.830). More precisely, the longer patients had diabetes as well as males were associated with a lower quality of life.

**Table 3:** Results of the full multivariable linear regression model adjusted for sex and age entering all predictors. The outcome was the average-weighted impact (AWI) score obtained in the Audit of Diabetes-Dependent Quality of Life (ADDQOL) questionnaire (Arab version)

Model	Predictors	Adjusted standardized
Full Model	(Constant)	−3.959 (−5.304 to −2.615)
	Age (continuous)	0.009 (−0.12 to 0.030)
	Sex Female Male	0.447 (0.064 to 0.830) ref
	Taking insulin currently Yes No	−0.256 (−0.689 to 0.177) ref
	Duration of diabetes (continuous)	−0.039 (−0.070 to −0.008)
	Diabetes complications Yes, at least one No	−0.254 (−0.690 to 0.182) ref

## Discussion

This study showed that a considerable proportion of patients with T2DM believed that T2DM had a negative impact on their quality of life. The results were compatible with a case–control study from Gaza in which diabetics expressed a poorer health-related quality of life than their healthy controls living in the same conditions [30]. Almost three-quarters of the patients in Ramallah and El-Bireh stated that their QoL would be better without diabetes. Similar percentages were found in a multinational study, including 5813 individuals conducted in nine different countries (Belgium, France, Germany, Greece, Italy, the Netherlands, Spain, Turkey, and the U.K.) [18]. The average weighted impact was −3.38. This AWI score was generally lower compared to those in other settings [19, 20, 28, 31–33], and it also differed significantly from the AWI (−1.69, *p* < 0.001) obtained in the multinational study [18]. This concurred with findings of a study that showed that the QoL of the Palestinian population ranked considerably lower than populations of the 17 pooled WHO International Field Trial countries using the WHO QoL questionnaire [10]. The difference in QoL could be explained by the protracted conflict, which reduces standards of diabetes care as well as limits the accessibility and affordability of medications [34].

It was observed that the life domain freedom to eat is adversely affected the most by T2DM. This is supported by other studies [12, 18–26, 28, 29, 32, 35]. In order to improve quality of life, diabetes management programs should focus on diet. This might include involving nutritionists more closely as they could give person-centered advice on diet. Critically, it is likely that this improves quality of life as patients has had a better AWI outcome if they had more information on T2DM. The aim of such diet programs should be to strengthen patient’s own capability to master challenges of the disease and to motivate the patient to make healthy choices.

The physical activity life domain was the second most adversely affected by T2DM. The relatively large perceived negative impact is only found in one other study using the ADDQoL-19 [29] and two studies using the ADDQoL-13 [12, 22]. Physical activity is an important preventive measure for disease progression as well as disease complications since it improves HbA1 control, lowers cardiovascular risk factors, is beneficial for weight loss, and improves overall wellbeing [36]. Diabetes management programs in Palestine should encourage physical activity and its long-lasting benefits in addition to healthy eating. This is also supported by authors of a study conducted in the West Bank, Palestine that compares five future policy scenarios for diabetes prevention. They concluded that diabetes can be largely prevented by policy interventions focusing on obesity reduction [2]. These interventions should be setting specific as cultural beliefs, lack of parks and walking areas play an important role against practising physical activity among Palestinians and this becomes exaggerated among diabetics (i.e. feeling uncomfortable due to polyuria).

Besides the freedom to eat and physical activity, people in Ramallah and El-Bireh expressed that their working life was impacted by diabetes greatly negatively as well. The people in Singapore also expressed that their working life was also considerably negatively impacted by diabetes [24]. Authors suggested that regular doctors visits interrupt work routines. Beyond this, sudden flare-ups of the disease might be interfering and discouraging. Work-life provided the *‘non-applicable’*-option in the ADDQoL questionnaire. This option was used by roughly two-thirds of the study participants. The reason for the high non-response rate in this domain might be that two-thirds of the study sample were women. In contrast, the working force participation rate of females above 15 years in Ramallah and El-Bireh is estimated to be only 11.8% [37]. Sex life had a non-response rate of 34.9%; this could be due to the cultural sensitivity of the topic.

A lower AWI score was associated with a worse diabetes-dependent QoL overview item. This coincided with findings from three other studies that the AWI is correlated more highly to the diabetes-dependent items than to the general QoL items [12, 18, 29].

Based on results of the regression analysis it was obtained that a lower QoL was associated with being male. In the QoL case–control study from Gaza, females showed lower QoL while in the general health-related QoL study from West Bank and Gaza, males showed lower QoL [10, 30]. Furthermore, there was an association between longer duration of diabetes and lower quality of life. Similar results that diabetes progresses over time were found elsewhere [22, 24, 25]. Duration of diabetes confounded the association of currently taking insulin negatively. Thus, the effect of taking insulin on AWI in the full model might be underestimated. On the predictor insulin, various studies showed that insulin treatment was associated with a more negative impact on QoL [18–27]. This perceived low QoL might be explained by the findings of a qualitative study from Jordan. T2DM patients considered insulin as the last means to treat diabetes and expressed fear of painful injections, which affects adherence. Authors associated taking insulin with complications and a later stage of the disease [38]. On complications, there was evidence that having complications lowers the quality of life [12, 18, 20, 21, 26, 28, 29]. This was not shown in this study. A review including patients with diabetes of Arab and Bedouin origin in ‘Israel’, yet, also found evidence of high rates of hospitalizations and micro and macrovascular complications. Beyond this, the review stated that financially difficulties are frequently expressed as cause for inadequate healthcare and consequent complications [39]. Another study stated that high numbers of patients, insufficient staff training, and inadequate infrastructure are the main impediments to the proper management of diabetes in Palestine. These authors recommended to standardize the different guidelines and have a comprehensive approach to management with provision of training for healthcare providers with respect to patient education and an improved system for keeping records of patients [40].

This study has the following limitations. Firstly, the study design was cross-sectional; thus, no temporality could be established. Secondly, participants were only recruited from primary healthcare clinics, not also from private clinics and households; hence, severe and bedridden cases might be underrepresented. Including this patient group, the QoL might be poorer. Thirdly, self-reported data, for instance, smoking habits, and physical activity could be subject to recall bias. Lastly, there was no full response of all 494 participants to the ADDQOL questionnaire. Despite these limitations, this study led to valuable insights into the impact of T2DM on quality of life in a population experiencing conflict and health care instabilities on a daily basis.

## Conclusion

T2DM patients from Ramallah and El-Bireh experienced diabetes as having a negative impact on their quality of life. Patients reported major constraints in food choices, physical activities, and work-life. They experienced a further decrease in quality of life if they had diabetes for a prolonged duration. These findings identified the demand for diabetes management programs tailored to the patients’ needs. Poor quality of life is not the only consequence of patient mismanagement but a challenge for health care organizations and governments to provide person-centered diabetes management according to International Guidelines on diabetes care [41]. For this, it might be useful to assess patients’ diabetes associated quality of life score as an outcome measure in order to motivate and enhance diabetes management and establish health policies that put patients at the center of diabetes care.

## Supplementary Information

Below is the link to the electronic supplementary material.

Supplementary Information 1 (DOCX 34 kb)

## Data Availability

Data available on request from corresponding author.
