# Supplementary material for: Quality of life of type 2 diabetes mellitus patients in Ramallah and al-Bireh Governorate–Palestine: a part of the Palestinian diabetes complications and control study (PDCCS)
Source: Qual Life Res. 2021 Mar 2;30(5):1407–16. doi: 10.1007/s11136-020-02733-w (PMC8068630; doi:10.1007/s11136-020-02733-w)
Supplement: Supplementary file 1 — Supplementary Information 1 (DOCX 34 kb) [file 11136_2020_2733_MOESM1_ESM.docx]

**Quality of Life of Type 2 Diabetes Mellitus patients in Ramallah and al Bireh Governorate – Palestine: a part of the Palestinian Diabetes Complications and Control Study (PDCCS)**

Anna Katharina Tietjen, Rula Ghandour, Nahed Mikki, Lars Jerdén, Jan W Eriksson, Margareta Norberg, Abdullatif Husseini

**Electronic Supplementary Material**

**Author information**

Anna Katharina Tietjen MPH (Corresponding author)

University of Lübeck

Ratzeburger Allee 160, 23562 Lübeck – Germany

anna.tietjen(at)student.uni-luebeck.de

Abdullatif Husseini, Ph.D., Professor

Epidemiology Unit, Institute of Community and Public Health

Said Khoury Building for Development Studies

Birzeit University, P.O.Box 14, Birzeit – Palestine
Tel: +970 2 2982019/20, Fax: +970 2 2982079

Abdullatif(at)birzeit.edu

Rula Ghandour MPH

Epidemiology Unit, Institute of Community and Public Health

Said Khoury Building for Development Studies

Birzeit University, P.O.Box 14, Birzeit – Palestine
Tel: +970 2 2982019/20, Fax: +970 2 2982079

[rghandour(at)birzeit.edu](mailto:rghandour@birzeit.edu)

Nahed Mikki MD, PhD

St. John Eye Hospital

Sheikh Jarrah – Jerusalem, P.O.Box 19960, Jerusalem 91198

Tel: +972 (0) 2 5828325 ext-224, Fax: +972(0) 2 5828327

Nahed.Mikki(at)sjeh.org, nahedmikki@gmail.com

Lars Jerdén, MD, Ph.D, Ass Professor

School of Education, Health and Social Studies

Dalarna University, 791 88 Falun, Sweden

Tel: +46 70 3040896

lars.jerden(at)regiondalarna.se

Jan W Eriksson, MD, Ph.D, Professor

Dept of Medical Sciences

Uppsala University, 751 85 Uppsala Sweden

jan.eriksson(at)medsci.uu.se

Margareta Norberg MD, Ph.D, Ass Professor

Department of Epidemiology and Public Health

Umeå University, 901 87 Umeå, Sweden

margareta.norberg(at)umu.se

**Acknowledgement**

We would like to thank the Palestinian Ministry of Health, the United Nations Works and Relief Agency, and the Palestinian Medical Relief Society, and the Palestinian Red Crescent Society for facilitating fieldwork, and the healthcare teams in the participating clinics for their cooperation. We also thank persons with diabetes for their participation and patience, as well as the field workers for their meticulous work. We also acknowledge the ADDQoL-19 questionnaire developers for their support in using this tool.

**Appendix 1:** Structure of the questionnaire audit of diabetes-dependent quality of life

*Table 1: Structure of the audit of diabetes-dependent quality of life (ADDQoL) instrument; table adapted from* [15, 30]

| **Audit of diabetes-dependent quality of life (ADDQoL)** |
| --- |
| **Overview items**   - General QoL: *In general, my present quality of life is: excellent / very good / good / neither good nor bad / bad / very bad / extremely bad*   - Score range^1^: +3 to –3 - Diabetes-dependent QoL: *If I did not have diabetes, my quality of life would be: very much better / much better / a little better / the same / worse*   - Score range^1^: –3 to +1   **Diabetes-specific questions**   - Impact of diabetes on a particular life domain: *If I did not have diabetes, my (life domain) would be:very much better / much better / a little better / the same / worse*   - Score range^1^: –3 to +1 - Importance of particular life domain: *My (life domain) is: very important / important / somewhat important / not at all important*   - Score range^2^: +3 to 0   **Life domains in ADDQoL**   - Family life^1^ - Friendship and social life - Close personal relationship^3^ - Sex life^3^ - Physical appearance - Physical health - Work/employment^3^ - Holiday^3^ - Leisure activities - Local or long-distance journeys - Self-confidence - Motivation - People’s reaction - Feelings about the future - Financial situation - Dependence on others - Living condition - Freedom to eat - Freedom to drink |
| ^1^More negative indicates a lower QoL. ^2^More positive indicates higher importance. ^3^Participants have not an applicable option of these domains. |

**Appendix 2:** The Cronbach’s α coefficient of internal consistency

*Table 2: The Cronbach’s α coefficient with each of the 19 items deleted for the Arabic ADDQoL (Overall: Cronbach’s α = 0.937)*

| **Domains** | **Scale Mean if Item Deleted** | **Scale Mean Variance if Item Deleted** | **Corrected item-total correlation** | **Cronbach’s alpha if item deleted** |
| --- | --- | --- | --- | --- |
| **Leisure activities** | -68,72 | 1696,75 | 0,68 | 0,933 |
| **Working life** | -67,35 | 1684,51 | 0,75 | 0,932 |
| **Journeys** | -67,99 | 1701,22 | 0,69 | 0,933 |
| **Holidays** | -68,35 | 1714,65 | 0,66 | 0,933 |
| **Physical activity** | -66,97 | 1692,49 | 0,69 | 0,933 |
| **Family life** | -67,81 | 1688,82 | 0,69 | 0,933 |
| **Friendship/social life** | -69,56 | 1723,31 | 0,65 | 0,934 |
| **Personal relationships** | -67,70 | 1691,48 | 0,68 | 0,933 |
| **Sex life** | -67,26 | 1714,89 | 0,61 | 0,934 |
| **Physical appearance** | -68,77 | 1704,65 | 0,66 | 0,933 |
| **Self confidence** | -68,25 | 1709,45 | 0,59 | 0,935 |
| **Motivation** | -67,66 | 1684,48 | 0,75 | 0,932 |
| **People's reaction** | -70,33 | 1751,86 | 0,59 | 0,935 |
| **Feeling about future** | -68,29 | 1694,72 | 0,67 | 0,933 |
| **Financial situation** | -69,15 | 1709,12 | 0,67 | 0,933 |
| **Living conditions** | -67,80 | 1686,20 | 0,72 | 0,932 |
| **Dependence on others** | -68,93 | 1778,10 | 0,41 | 0,938 |
| **Freedom to eat** | -66,82 | 1758,17 | 0,47 | 0,937 |
| **Freedom to drink** | -66,97 | 1737,15 | 0,55 | 0,936 |

Table 2 shows an analysis of reliability using the Cronbach’s α coefficient for internal consistency. The overall Cronbach’s α coefficient was 0.937. The Cronbach’s α coefficient was not increased deleting the 19 items expect from the item “Dependence on others”. For this item, the Cronbach’s α coefficient was only increased slightly to 0.938. Nevertheless, this item was retained. “Dependence on others” and “freedom to eat” have the smallest corrected total-item correlation (0.41 and 0.47, respectively) which means those correlate the least with the overall questionnaire score.

**Appendix 3:** Unforced Factor Analysis using Varimax including all items

*Table 3: Unforced Factor Analysis using Varimax including all 19 items loading greater than the factor 0.4*

| **Domains** | **Factors** | | |
| --- | --- | --- | --- |
|  | 1 | 2 | 3 |
| Leisure activities | 0.564661 |  |  |
| Working life | 0.770183 |  |  |
| Journeys | 0.780321 |  |  |
| Holidays | 0.711108 |  |  |
| Physical activity | 0.750357 |  |  |
| Family life | 0.609211 |  |  |
| Friendship/social life |  | 0.701817 |  |
| Personal relationships | 0.655021 | 0.407896 |  |
| Sex life | 0.709971 |  |  |
| Physical appearance |  | 0.693199 |  |
| Self confidence |  | 0.652391 |  |
| Motivation | 0.664434 | 0.433044 |  |
| People's reaction |  | 0.782543 |  |
| Feeling about future | 0.455872 | 0.533868 |  |
| Financial situation | 0.528667 | 0.411584 |  |
| Living conditions | 0.672618 | 0.402775 |  |
| Dependence on others |  | 0.628956 |  |
| Freedom to eat |  |  | 0.900723 |
| Freedom to drink |  |  | 0.877025 |

Unforced Factor Analysis using Varimax generated three factors with eigenvalues above 1. The three factors explain 61.801% of the variance. Twelve domains loaded greater than 0.4 on factor 1, ten on factor 2, and two on factor 3. The two items loaded on factor 3 are both concerned with dietary habits.

**Appendix 4:** Impact, importance and impact weighted by the importance for ADDQoL

*Table 4: Distribution of Audit Diabetes Dependent Quality of life-19 (ADDQOL) Arab version results for specific life domain items: Impact, mean(SD): very much greater to less, scored from (-3 to 1); Importance, mean(SD): very important to not at all important, scored from (3 to 0), weighted impact by importance, mean(SD), median(Range), scored from -9 to 3(n = 494)*

| **Specific life domain** | **N** | **Impact** | | **Importance** | **Impact weighted by the importance**  **ADDQoL Score** | | |
| --- | --- | --- | --- | --- | --- | --- | --- |
|  |  | Mean(SD) | Rank | Mean(SD) | Mean(SD) | Median(Range) | Rank |
| Freedom to eat | 485 | -1.93(1.07) | 1 | 2.23(0.82) | -4.60(3.21) | -4.0(-9 to 2) | 1 |
| Physical activities | 492 | -1.76(1.09) | 3 | 2.52(0.57) | -4.53(3.16) | -4.0(-9 to 3) | 2 |
| Working life^1^ | 160 | -1.66(1.15) | 5 | 2.55(0.58) | -4.32(3.27) | -4.0(-9 to 0) | 3 |
| Freedom to drink | 486 | -1.80(1.08) | 2 | 2.15(0.86) | -4.16(3.15) | -4.0(-9 to 1) | 4 |
| Motivation | 487 | -1.67(1.06) | 4 | 2.38(0.59) | -4.08(3.00) | -4.0(-9 to 0) | 5 |
| Family life^1^ | 486 | -1.44(1.14) | 10 | 2.69(0.52) | -3.86(3.30) | -4.0(-9 to 0) | 6 |
| Living conditions | 486 | -1.50(1.10) | 8 | 2.41(0.65) | -3.77(3.08) | -4.0(-9 to 0) | 7 |
| Sex life^1^ | 309 | -1.62(1.15) | 6 | 2.13(0.86) | -3.75(3.29) | -4.0(-9 to 0) | 8 |
| Journeys | 491 | -1.61(1.09) | 7 | 2.11(0.82) | -3.57(3.00) | -4.0(-9 to 3) | 9 |
| Personal relationships^1^ | 391 | -1.36(1.15) | 13 | 2.52(0.61) | -3.52(3.26) | -4.0(-9 to 0) | 10 |
| Depend on others | 485 | -1.38(1.12) | 12 | 2.52(0.73) | -3.49(3.17) | -3.0(-9 to 3) | 11 |
| Feelings about the future | 483 | -1.43(1.15) | 11 | 2.19(0.84) | -3.40(3.17) | -4.0(-9 to 0) | 12 |
| Self-confidence | 487 | -1.30(1.16) | 14 | 2.57(0.53) | -3.38(3.27) | -4.0(-9 to 3) | 13 |
| Holidays^1^ | 421 | -1.46(1.15) | 9 | 1.95(0.95) | -3.06(3.01) | -3.0(-9 to 3) | 14 |
| Physical appearance | 486 | -1.24(1.17) | 16 | 2.37(0.72) | -3.06(3.18) | -2.5(-9 to 2) | 15 |
| Leisure activities | 494 | -1.28(1.20) | 15 | 1.96(0.95) | -2.76(3.08) | -2.0(-9 to 3) | 16 |
| Friendship/Social life | 488 | -1.04(1.15) | 17 | 2.30(0.76) | -2.49(3.05) | 0.0(-9 to 0) | 17 |
| Financial situation | 486 | -0.86(1.07) | 18 | 2.42(0.71) | -2.17(2.87) | 0.0(-9 to 0) | 18 |
| People’s reaction | 487 | -0.51(0.93) | 19 | 2.26(0.81) | -1.19(2.40) | 0.0(-9 to 3) | 19 |

*1 Domains with not applicable (NA) option*
